# Genome-Wide Diversity in the Levant Reveals Recent Structuring by Culture

**DOI:** 10.1371/journal.pgen.1003316

**Published:** 2013-02-28

**Authors:** Marc Haber, Dominique Gauguier, Sonia Youhanna, Nick Patterson, Priya Moorjani, Laura R. Botigué, Daniel E. Platt, Elizabeth Matisoo-Smith, David F. Soria-Hernanz, R. Spencer Wells, Jaume Bertranpetit, Chris Tyler-Smith, David Comas, Pierre A. Zalloua

**Affiliations:** 1 Institut de Biologia Evolutiva (CSIC–UPF), Departament de Ciències de la Salut i de la Vida, Universitat Pompeu Fabra, Barcelona, Spain; 2 The Lebanese American University, Chouran, Beirut, Lebanon; 3 The Wellcome Trust Centre for Human Genetics, University of Oxford, Oxford, United Kingdom; 4 INSERM UMRS872, Centre de Recherche des Cordeliers, Paris, France; 5 Broad Institute of Harvard and MIT, Cambridge, Massachusetts, United States of America; 6 Department of Genetics, Harvard Medical School, Boston, Massachusetts, United States of America; 7 Bioinformatics and Pattern Discovery, IBM T. J. Watson Research Centre, Yorktown Heights, New York, United States of America; 8 Allan Wilson Centre for Molecular Ecology and Evolution and Department of Anatomy, University of Otago, Dunedin, New Zealand; 9 The Genographic Project, National Geographic Society, Washington, D.C., United States of America; 10 The Wellcome Trust Sanger Institute, Wellcome Trust Genome Campus, Hinxton, United Kingdom; 11 Harvard School of Public Health, Boston, Massachusetts, United States of America; Dartmouth College, United States of America

## Abstract

The Levant is a region in the Near East with an impressive record of continuous human existence and major cultural developments since the Paleolithic period. Genetic and archeological studies present solid evidence placing the Middle East and the Arabian Peninsula as the first stepping-stone outside Africa. There is, however, little understanding of demographic changes in the Middle East, particularly the Levant, after the first Out-of-Africa expansion and how the Levantine peoples relate genetically to each other and to their neighbors. In this study we analyze more than 500,000 genome-wide SNPs in 1,341 new samples from the Levant and compare them to samples from 48 populations worldwide. Our results show recent genetic stratifications in the Levant are driven by the religious affiliations of the populations within the region. Cultural changes within the last two millennia appear to have facilitated/maintained admixture between culturally similar populations from the Levant, Arabian Peninsula, and Africa. The same cultural changes seem to have resulted in genetic isolation of other groups by limiting admixture with culturally different neighboring populations. Consequently, Levant populations today fall into two main groups: one sharing more genetic characteristics with modern-day Europeans and Central Asians, and the other with closer genetic affinities to other Middle Easterners and Africans. Finally, we identify a putative Levantine ancestral component that diverged from other Middle Easterners ∼23,700–15,500 years ago during the last glacial period, and diverged from Europeans ∼15,900–9,100 years ago between the last glacial warming and the start of the Neolithic.

## Author Summary

Population stratification caused by nonrandom mating between groups of the same species is often due to geographical distances leading to physical separation followed by genetic drift of allele frequencies in each group. In humans, population structures are also often driven by geographical barriers or distances; however, humans might also be structured by abstract factors such as culture, a consequence of their reasoning and self-awareness. Religion in particular, is one of the unusual conceptual factors that can drive human population structures. This study explores the Levant, a region flanked by the Middle East and Europe, where individual and population relationships are still strongly influenced by religion. We show that religious affiliation had a strong impact on the genomes of the Levantines. In particular, conversion of the region's populations to Islam appears to have introduced major rearrangements in populations' relations through admixture with culturally similar but geographically remote populations, leading to genetic similarities between remarkably distant populations like Jordanians, Moroccans, and Yemenis. Conversely, other populations, like Christians and Druze, became genetically isolated in the new cultural environment. We reconstructed the genetic structure of the Levantines and found that a pre-Islamic expansion Levant was more genetically similar to Europeans than to Middle Easterners.

## Introduction

The Levant is a geographical area in the eastern Mediterranean region bounded by Anatolia, Egypt, and the Arabian Desert. It includes Lebanon, Syria, Jordan, Israel, Palestine, and often Cyprus and historical Armenia. The region has been central to human cultural development, embracing the earliest civilizations, agricultural communities, and the rise of the first urban cities. The genetic diversity based on uniparental markers (i.e. Y-chromosome and mtDNA) of the Levantine populations shows a strong correlation with geography [1] and religion [2]–[4]. It has been suggested that the Islamic expansion from the Arabian Peninsula beginning in the 7th century CE introduced lineages typical of this Peninsula into those who subsequently became Lebanese Muslims, whereas the Crusader activity in the 11^th^–13^th^ centuries CE introduced western European lineages into Lebanese Christians [5]. This recent differential penetration of exogenous Y-chromosome lineages into the Lebanese has probably been maintained by limited admixture between the religious groups, resulting in population stratifications in the present-day populations. However, it is not yet known if those structures are genome-wide and if they extend beyond Lebanese borders. Genome-wide surveys in the Levant are limited and most of our knowledge comes from studies assessing the relationship of Diaspora Jewish groups to a Levantine/Middle Eastern origin [6], [7]. These studies show that the Jews form a distinctive cluster in the Middle East, and it is not known whether the factors driving this structure would also involve other groups in the Levant. For example, would the Druze from Mount Lebanon have the same genome-wide diversity as the Druze from Mount Carmel, and would the predominantly Muslim populations in the Levant from Syria, Palestine, and Jordan have more genetic similarities to the populations of the Arabian Peninsula (Saudis, Yemenis) than would other non Muslims Levantines have? A recent study by Moorjani et al. [8], estimated that Jewish admixture with African genes ended much earlier (∼75 generations ago) than other Levantines (Muslims) (∼32 generations ago). However, it is not known if this different admixture history is the result of out-migration from the region and the discontinued gene flow from neighboring populations or if it is a result of cultural isolation in a predominantly Christian (∼100–650 CE) and later Muslim (∼650 CE-present) environment. Would today's Christians from the Levant also show older dates for cessation of African admixture than other Levantines, reflecting cultural/genetic isolation from their surrounding neighbors? By exploring the genetic isolation of populations like the Christians and Druze, it would then be possible to assess the pre-Islamic genetic structure of the Levantines and accurately construct the genetic relationships with neighboring populations.

In this study we analyze newly-generated genome-wide data from Lebanon in addition to individuals from 48 published global populations [7], [9]. We aim to assess the genome-wide genetic relationships of the Levantines and to resolve previous uncertainties about population structure in the Levant region. We pay particular attention to cultural influences on genetic structure, and explore the consequences of more than 2,000 years of cultural differentiation on the genetic composition of modern Levantines.

## Results/Discussion

### Genome-wide structure of the Lebanese

**Figure 1:**
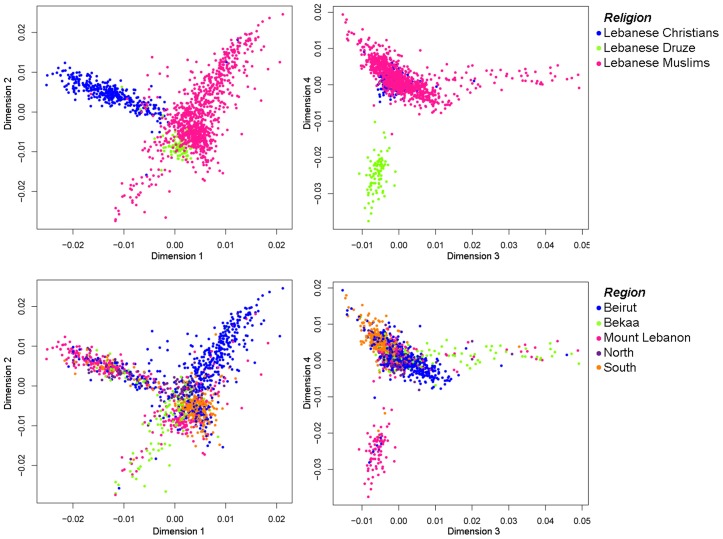
Multidimensional scaling of >240K SNPs in 1,341 Lebanese samples showing the first four dimensions. The SNPs were pruned from >500,000 SNPs excluding r^2^>0.4. The samples were classified by their religion or region of origin.

A multidimensional scaling (MDS) plot based on the identity-by-state (IBS) matrix shows strong stratification in Lebanon by religion, with separate clusters for Christians, Muslims, and Druze, irrespective of their geographic origin (Figure 1). The results suggest endogamous practices among the religious groups of Lebanon within a small geographical area not exceeding 10,452 km^2^ (half the size of the state of New Jersey or one third the size of Belgium). Christianity in Lebanon dates back to the first century CE, whereas Islam was brought to the Levant through the Islamic expansions in 635 CE. In 986 CE, the Druze faith developed as a movement within Islam, and from 1030 AD, a person could only be Druze if born Druze. This correlation of genetic structure within Lebanon with cultural traits was previously described by Haber et al. [3] based on the religious structuring of Y-chromosomal variation within Lebanon, but here we see it is genome-wide. In order to assess the proportion of putative ancestral components in the Lebanese, an unsupervised clustering method (ADMIXTURE) [10] was applied to the Lebanese dataset (Figure S1A). At *K* = 2, which showed the lowest cross-validation error (Figure S1B), Christians present one major component (∼82% on average per individual), which is also found in Druze and in lower frequencies in Muslims; in contrast, the second component is almost exclusive to Muslims with a lower representation in Druze. At *K* = 3 and *K* = 4, new components most abundant in Lebanese Muslims are shown, probably reflecting recent admixture after the split from the other Lebanese groups.

### Genome-wide structure of the Levantines

**Figure 2:**
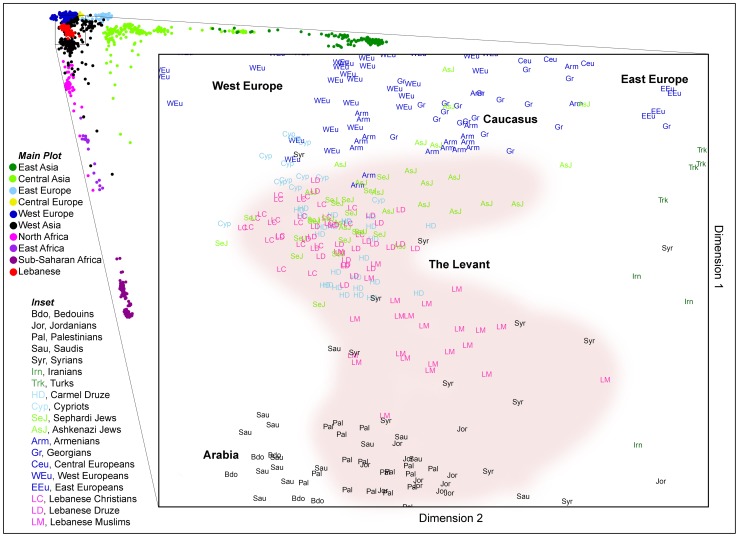
Multidimensional scaling of >240K SNPs showing the top two dimensions. Main plot shows global diversity using 50 populations. Inset shows Levantine populations in their regional and religion context. The Levant region includes Lebanon, Syria, Jordan, Israel, Palestine, and often Cyprus and historical Armenia. The Levantine core cluster is shaded in pink.

In order to assess the population structure of Levantine populations more generally, an MDS (Figure 2) and a normalized principle component analysis (PCA) (Figure S2) plots with 48 additional Old World populations (Table S1) were built. Only 25 randomly selected samples from each Lebanese group were used in order to avoid population size biases (Figure S3). The plots reveal a Levantine structure not reported previously: Lebanese Christians and all Druze cluster together, and Lebanese Muslims are extended towards Syrians, Palestinians, and Jordanians, which are close to Saudis and Bedouins. Ashkenazi Jews are drawn towards the Caucasus and Eastern Europe, reflecting historical admixture events with Europeans, while Sephardi Jews cluster tightly with the Levantine groups. These results are consistent with previous studies reporting higher European genome-wide admixture in Ashkenazi Jews compared with other Jews [11] and higher Y-chromosomal gene flow to Lebanese Muslims from the Arabian Peninsula compared with other Lebanese [5].

### Inferences of population relations from haplotypes

**Figure 3:**
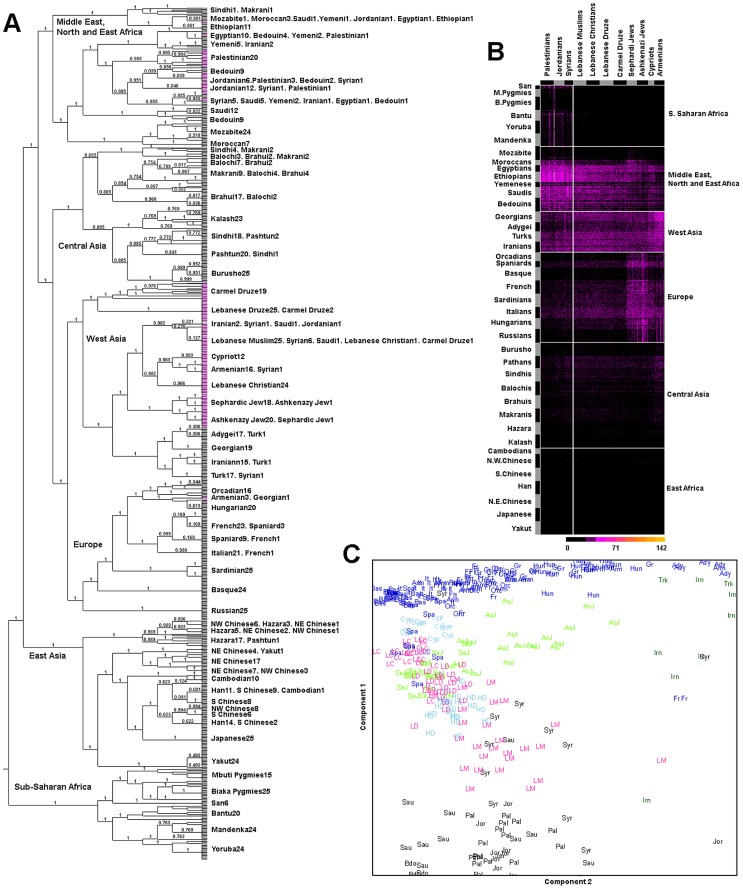
Population relationships from genome-wide haplotypes. A) Each tip of the tree corresponds to an individual; numbers of individuals are shown next to their population name at the tip of the branches. Numbers on branches show partition posterior probability. The Levantine populations' tips are highlighted in pink. B) Raw coancestry matrix shows relationships between the Levantines and the world populations. Intensity of the colors reflects the number of haplotype chunks donated to the Levantines. The vertical line is a visual aid to reflect the Levantine split observed in the tree. Horizontal lines distinguish the major geographic regions. C) Principal component analysis using the world coancestry matrix, figure is magnified on West Asia.

The previous analyses are based on linkage disequilibrium (LD) pruned data (r^2^<0.4) since LD can bias cluster analysis. However, identification of haplotypes shared between groups is a valuable tool to infer population history events [12]–[15]. Thus, we phased our data and generated a coancestry matrix using ChromoPainter [16] which reconstruct the haplotype of every individual using the haplotypes of each of the other individuals as possible donors. ChromoPainter computes a similarity measure which is the number of haplotype “chunks” used to reconstruct the recipient individual from each donor individual. We then used fineSTRUCTURE [16] which employ model-based Bayesian clustering to construct a tree that infer population relationships and similarities using ChromoPainter's coancestry matrix. The population tree (Figure 3A) splits Levantine populations in two branches: one leading to Europeans and Central Asians that includes Lebanese, Armenians, Cypriots, Druze and Jews, as well as Turks, Iranians and Caucasian populations; and a second branch composed of Palestinians, Jordanians, Syrians, as well as North Africans, Ethiopians, Saudis, and Bedouins. The tree shows a correlation between religion and the population structures in the Levant: all Jews (Sephardi and Ashkenazi) cluster in one branch; Druze from Mount Lebanon and Druze from Mount Carmel are depicted on a private branch; and Lebanese Christians form a private branch with the Christian populations of Armenia and Cyprus placing the Lebanese Muslims as an outer group. The predominantly Muslim populations of Syrians, Palestinians and Jordanians cluster on branches with other Muslim populations as distant as Morocco and Yemen. It should be noted here that the results depend significantly on populations included in the analysis as well as recent admixture events, and so should be treated as an approximate guide to similarity, rather than a full population history.

ChromoPainter's coancestry matrix (Figure 3B, Figure S4) shows the haplotype chunks donated from the world populations to the Levantines and shows that Jordanians, Palestinians, and Syrians receive more chunks from sub-Saharan Africans and from Middle Easterners compared with other Levantines. We explored the sub-Saharan/Middle Eastern gene flow to the Levantines further by employing a previously developed method (ROLLOFF) [8] that estimates the time since admixture with sub-Saharan African genes using the rate of exponential decline of admixture LD. Previous simulations [8] showed that bias from ROLLOFF estimates is removed with increased sample size, so we used the entire Lebanese religious subgroups after carrying out a rigorous outlier removal based on PCA [17] and keeping the main core clusters (336 Christians, 85 Druze, 747 Muslims) (Text S1). We found that Christians have the oldest admixture dates (2,375-2,025 years ago, y.a) with bounds coinciding with the decline of Phoenicia and the control of the region by the Hellenistic rulers. The time since the observed Druze admixture (1,275-1,025 y.a) closely precedes the development of the Druze faith and their divergence from other Muslims. The Muslims appear to have maintained contact with populations carrying sub-Saharan genes until 675-625 y.a, which overlaps with the rise of the Ottoman Empire and formation of a semi-autonomous state in Lebanon. Historical events coinciding with our observed admixture dates are some of the examples of population processes and demographic events that were occurring during this period in the Levant. These historical events, in addition to cultural adoptions and transitions, may have contributed to the differences among the religious groups through facilitating or restricting contact with other Middle Easterners carrying the sub-Saharan genes. It should also be noted here that ROLLOFF estimates dates assuming instantaneous mixture, without distinguishing between the patterns expected for instantaneous admixture and continuous gene flow. Previous simulations [8] show that for continuous gene flow, the dates from ROLLOFF reflect the average of mixture dates over a range of times, hence the date should be interpreted only as an average number.

The principal component plot performed with the coancestry matrix (Figure 3C, Figure S5) is similar to the pattern seen in West Asia with the MDS and PCA analysis based on LD-pruned SNPs.

### Admixture analysis and ancestral population divergence

In order to identify and quantify the ancestral components in the Levantines, an ADMIXTURE analysis [10] was performed with Old World samples (Figure S6A).

ADMIXTURE requires the assignment of a specific population number (K). We chose to assign a *K* = 10 (Figure S6, Table S3) since it captures many of the population structures identified by fineSTRUCTURE, particularly the formation of separate ancestral components for Levantines and Middle Easterners. ADMIXTURE's cross-validation (Figure S6B) shows that *K* = 8 has the lowest cross-validation (CV) error, however the CV effectiveness in predicting the “truth” *K* can be challenged when considering closely related populations [18]–[20]. Therefore, in this analysis we use the ChromoPainter/fineSTRUCTURE pipeline to identify fine populations subdivisions without the drawback of specifying a *K* value [16], [20], and use ADMIXTURE to estimate the genetic distances between the ancestral components independent of subsequent admixture events.

**Figure 4:**
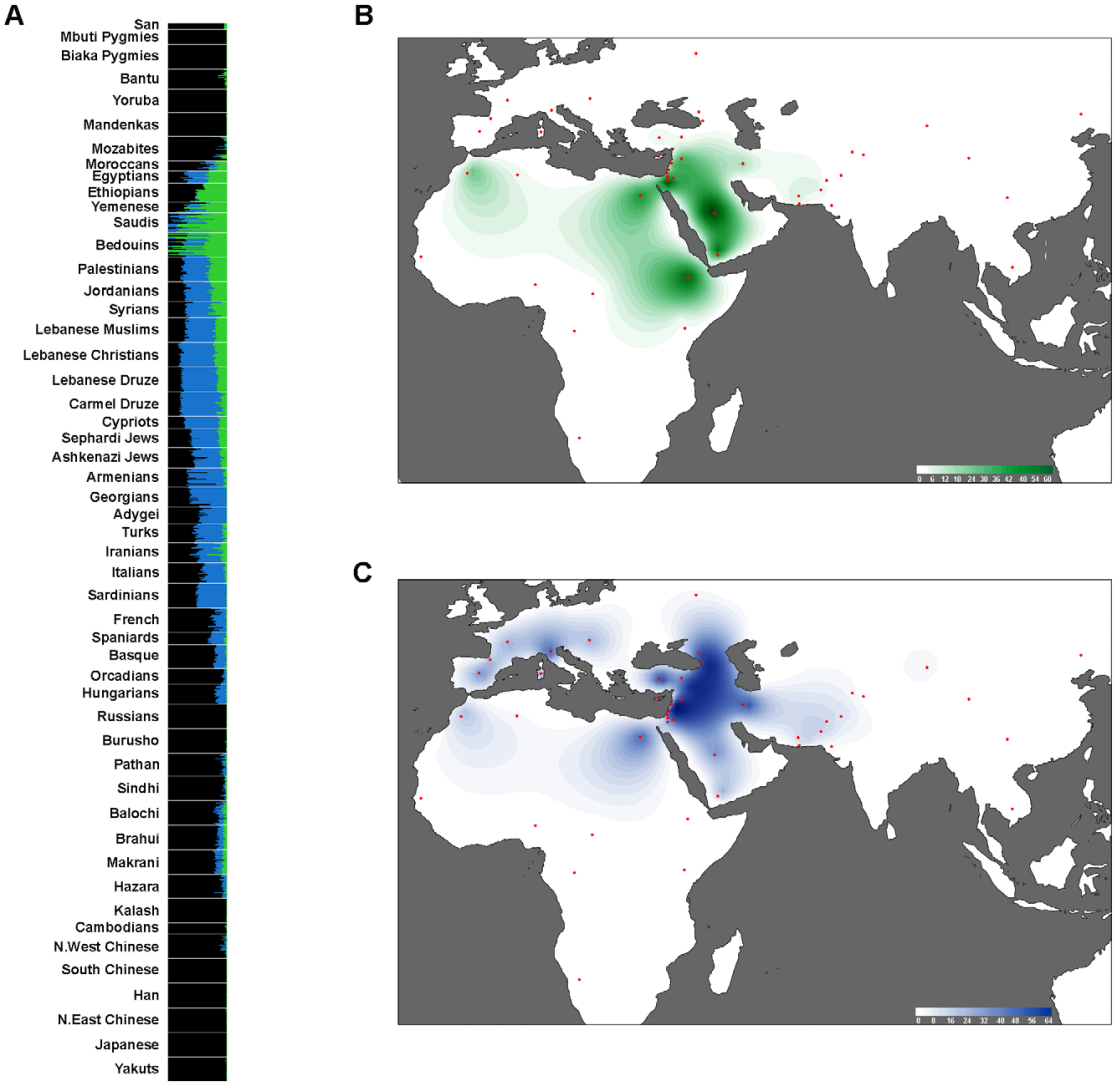
Comparisons of the Levantine and Middle Eastern modal components. A) ADMIXTURE analysis based on 10 constructed ancestral components, with only the Levantine and Middle Eastern components highlighted. B) Frequency of the Middle Eastern component in world populations. C) Frequency of the Levantine component in world populations. Intensity of the colors reflects the frequency of a component in the plotted populations. Maps were produced using a weighted average interpolating algorithm, and therefore should be used as a guide rather than a precise representation of the frequency distribution.

ADMIXTURE identifies at *K* = 10 an ancestral component (light green) with a geographically restricted distribution representing ∼50% of the individual component in Ethiopians, Yemenis, Saudis, and Bedouins, decreasing towards the Levant, with higher frequency (∼25%) in Syrians, Jordanians, and Palestinians, compared with other Levantines (4%–20%). The geographical distribution pattern of this component (Figure 4A, 4B) correlates with the pattern of the Islamic expansion, but its presence in Lebanese Christians, Sephardi and Ashkenazi Jews, Cypriots and Armenians might suggest that its spread to the Levant could also represent an earlier event. Besides this component, the most frequent ancestral component (shown in dark blue) in the Levantines (42–68%) is also present, at lower frequencies, in Europe and Central Asia (Figure 4A, 4C). We found that this Levantine component is closer to the European component (dark green) (*F_ST_ = *0.035) than to the Arabian Peninsula/East Africa component (light green) (*F_ST_* = 0.046). Our estimates show that the Levantine and the Arabian Peninsula/East African components diverged ∼23,700-15,500 y.a., while the Levantine and European components diverged ∼15,900-9,100 y.a. We note here that our divergence time estimates are based on the assumption that “effective population sizes” have not significantly changed overtime. We make this assumption, and obtain divergence times from genetic data which appear to coincide well with archeology.

The estimated time of divergence between the Levantine component and other Middle Easterners overlaps with evidence from archeological findings of a major cultural development in the Levant during the early Epipaleolithic period (23,000-14,500 y.a) [21]. The period of climatic warming after the Last Glacial Maximum (∼26,000-19,900 y.a) in the Levant was characterized by the spread of the microlithic technologies and the appearance of highly mobile populations between the Sinai Peninsula and southern Turkey. This Early Epipaleolithic phase formed a cultural continuity with the last Epipaleolithic phase, immediately preceding the appearance of the Natufian culture and the development of sedentism [22]. Our time estimate of divergence between the Levantine and European components (∼15,900-9,100 y.a) overlaps with the transition to agriculture in the Levant ∼11,000 y.a but is also slightly earlier than the proposed expansion to Europe starting at ∼9,000 y.a. [23]–[25]. In agreement with this, a recent study of complete mtDNA sequences also proposed earlier expansion dates (19,000-12,000 y.a) of certain female lineages from the Near East to Europe [26]. These results suggest that population migration to Europe from the Near East could have started after the LGM warming and continued until the Neolithic. In addition, these results show that the modern European genetic component is more recent than would be expected from a component that developed from the initial peopling of Europe in the Upper Paleolithic ∼40,000 y.a.

### Conclusions

From the first ventures out of Africa, to admixture with archaic humans, to the earliest Neolithic transition, the developments in the Levant have marked the history of modern humans. However, the Levant had been underrepresented in genome-wide studies and little is known about its population structure. In this study, we show a multilayered history of the Levantines with multiple components that might be traced to different historical population events. We propose that the Levant and Middle Eastern modal components diverged after the LGM during the early Epipaleolithic period, which was characterized by behavioral variability and innovations accompanied by major life-style and technological changes in the Levant [21], [27], [28]. We also show that the Levantines and Europeans diverged between the last glacial warming and the start of the Neolithic age.

Finally, we show that although population movements and expansions during the Epipaleolithic marked the emergence of a Levantine component and made the Levantines genetically similar, recent cultural developments, such as the founding and spread of major world religions, have had a strong impact on population stratifications in the Levant.

Populations like the Levantines, where geography is not the only major correlate of genetic variation, are unusual. In addition to their importance in understanding human evolution and history, these unusual stratifications can be hard to control in association studies for mapping complex disease susceptibilities and therefore require particular attention.

## Materials and Methods

### Subjects, genotyping, and comparative datasets

Samples were collected from 1,341 Lebanese subjects with informed consent approved by the IRB of the Lebanese American University. Genotyping was performed on Illumina 610K or 660K bead arrays. PLINK [29] was used for data management and quality control. Genotyping success rate was set to 99%, sex-linked and mitochondrial SNPs removed, keeping 505,859 SNPs. After LD pruning (excluding r2>0.4) 244,919 SNPs remained. 75 Lebanese samples (Figure S3, Table S1) were selected through a stratified random sampling taking into consideration the distribution of the religious groups in Lebanon and merged with 994 samples from literature representing 48 populations (Table S2). The selected Lebanese data set is available at: bhusers.upf.edu/~mhaber/PLOS/

### Population structure

#### Multidimensional scaling and principle component analysis

The *N x N* matrix of the genome-wide IBS pairwise distances was constructed using PLINK on pruned SNPs. The MDS was performed in the R environment [30].

PCA was performed using *smartpca*, part of the EIGENSOFT 3.0 package [17].

#### Inference of population relations from haplotypes

Samples (505,859 SNPs) were phased with SHAPEIT [31] using as a reference the HapMap3 genetic maps [32]. A coancestry matrix was constructed using ChromoPainter [16] with the default settings. FineSTRUCTURE [16] was used to perform an MCMC iteration on the coancestry matrix generated by ChromoPainter using 10,000,000 burnin and runtime and 10,000 MCMC samples. A tree was built using fineSTRUCTURE which starts with the maximum a posteriori state by taking the MCMC iteration with the highest observed posterior likelihood. Starting from this initial partition, additional hill-climbing moves are then performed, successively merging and splitting populations, and identifying the merges that further improve the Posterior probability, generating a bifurcating tree of relationships amongst these populations.

#### Estimating time since admixture

ROLLOFF analysis [8] was performed to estimate the time since mixture with sub-Saharan African genes. The analysis was carried out using Georgians and Ethiopians as the reference populations and a generation time of 25 years. The choice of the reference populations is shown not to be critical (Text S1) as the use of Sardinians and West Africans (Yoruba), as reference populations, produced qualitatively similar results.

#### Admixture analysis

The clustering algorithm ADMIXTURE [10] was used on pruned SNPs and different number of ancestral populations were considered. The plots were visualized using R.

*F_STs_* were calculated using ADMIXTURE at *K* = 10. In order to map our *F_ST_* estimates into population divergence dates some information about population demography is needed. Li et al. [9] provide an estimate of “effective population size” for 53 human populations which can be interpreted as the mean time to a common ancestor for genetic material from 2 aligned chromosomes from the population. We use this information to estimate the divergence time between the ancestral components using the relationship [33] between *F_ST_* and the effective population size *Ne* (using the range for Europeans (5,379–8,677) [9] and Middle Easterners (7,006–9,505) [9], using 25 years generation time.

Contour maps showing the distribution of the ancestral components were generated using Surfer 8 (Golden Software) implementing the Kriging method.

## Supporting Information

Figure S1 Lebanon religion groups structure inferred by ADMIXTURE analysis of >240K autosomal SNPs. A) Each horizontal line represents ancestry probabilities of an individual in the 2–4 constructed ancestral populations. B) Cross-validation plot for the Lebanese dataset.
(TIF)

Figure S2 Principle component analysis of >240K SNPs showing the top two components. A) Plot shows global diversity using 50 populations. B) Magnification of West Asia region showing the Levantine populations in their regional and religion context.
(TIF)

Figure S3 Stratified random sampling of 75 Lebanese samples. A) 25 samples from each of the three main religion groups in Lebanon were randomly chosen from the 1,341 samples illustrated in Figure 1. B) Map of Lebanon showing the distribution of the samples.
(TIF)

Figure S4 Raw coancestry matrix shows relationships between the Levantines and the world populations. A) Intensity of the colors reflects the number of haplotype chunks donated to the Levantines. The vertical line is a visual aid to reflect the Levantine split observed in the tree. Horizontal lines distinguish the major geographic regions. B) coancestry matrix with an alternative color scale.
(TIF)

Figure S5 Principle component analysis generated with fineSTRUCTURE using ChromoPainter's coancestry matrix showing the top two components. A) Plot shows global diversity using 50 populations. B) Magnification of West Asia region showing the Levantine populations in their regional and religion context.
(TIF)

Figure S6 World population structure inferred by ADMIXTURE analysis of >240K autosomal SNPs. A) Each horizontal line represents ancestry probabilities of an individual in 2–10 constructed ancestral populations. Levantine population names are shown in blue. B) Cross-validation plot for the world dataset.
(TIF)

Table S1 Location of the 75 Lebanese samples used in the comparative analyzes.
(XLS)

Table S2 Populations selected for this study.
(XLS)

Table S3 Ancestry probabilities of individuals considering 10 ancestral populations. Highlighted cells indicate individuals have >60% of one component. Standard errors were estimated using 200 bootstrap replicates.
(XLS)

Text S1 Description of the ROLLOFF analysis.
(PDF)
