# Supplementary figures and images for: Genome-Wide Diversity in the Levant Reveals Recent Structuring by Culture
Source: PLoS Genet. 2013 Feb 28;9(2):e1003316. doi: 10.1371/journal.pgen.1003316 (PMC3585000; doi:10.1371/journal.pgen.1003316)

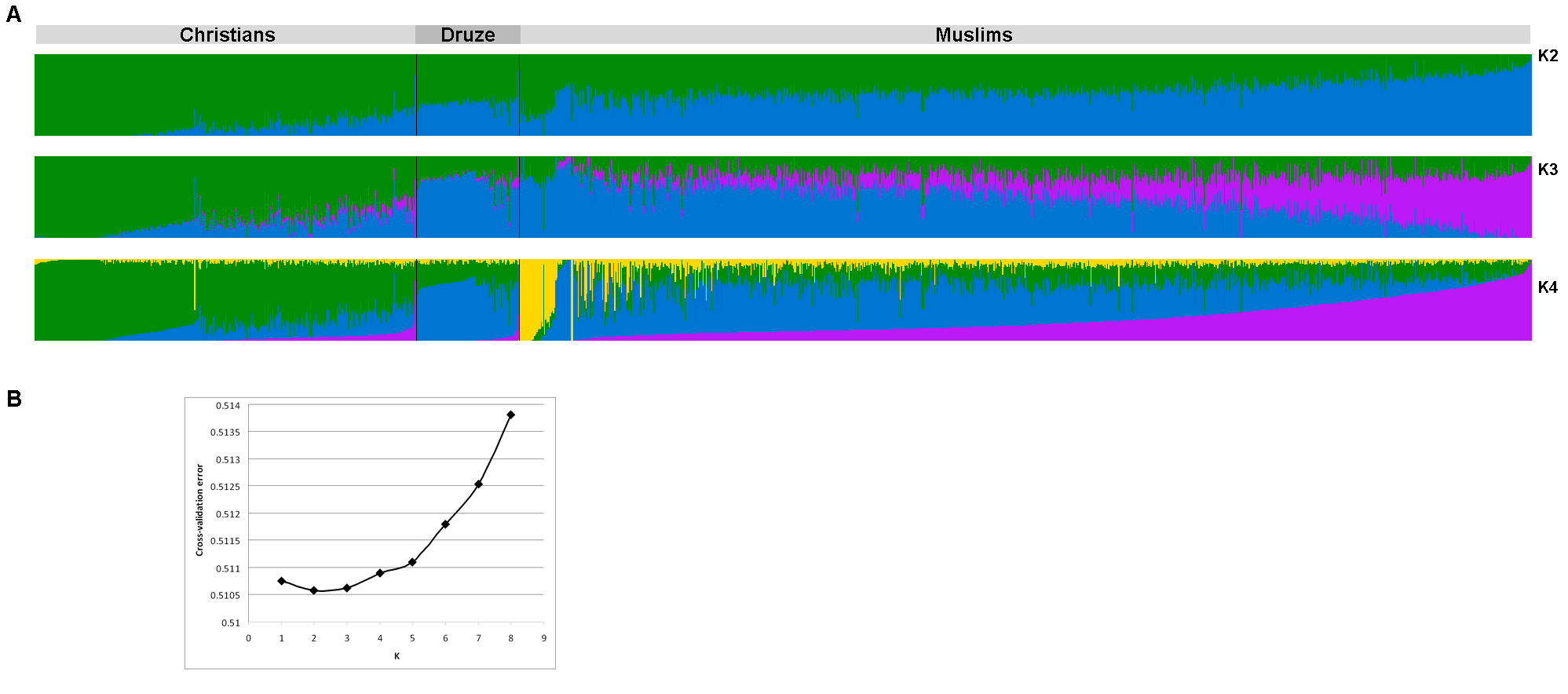

Supplement: Figure S1 — Lebanon religion groups structure inferred by ADMIXTURE analysis of >240K autosomal SNPs. A) Each horizontal line represents ancestry probabilities of an individual in the 2–4 constructed ancestral populations. B) Cross-validation plot for the Lebanese dataset. (TIF) [file pgen.1003316.s001.tif]

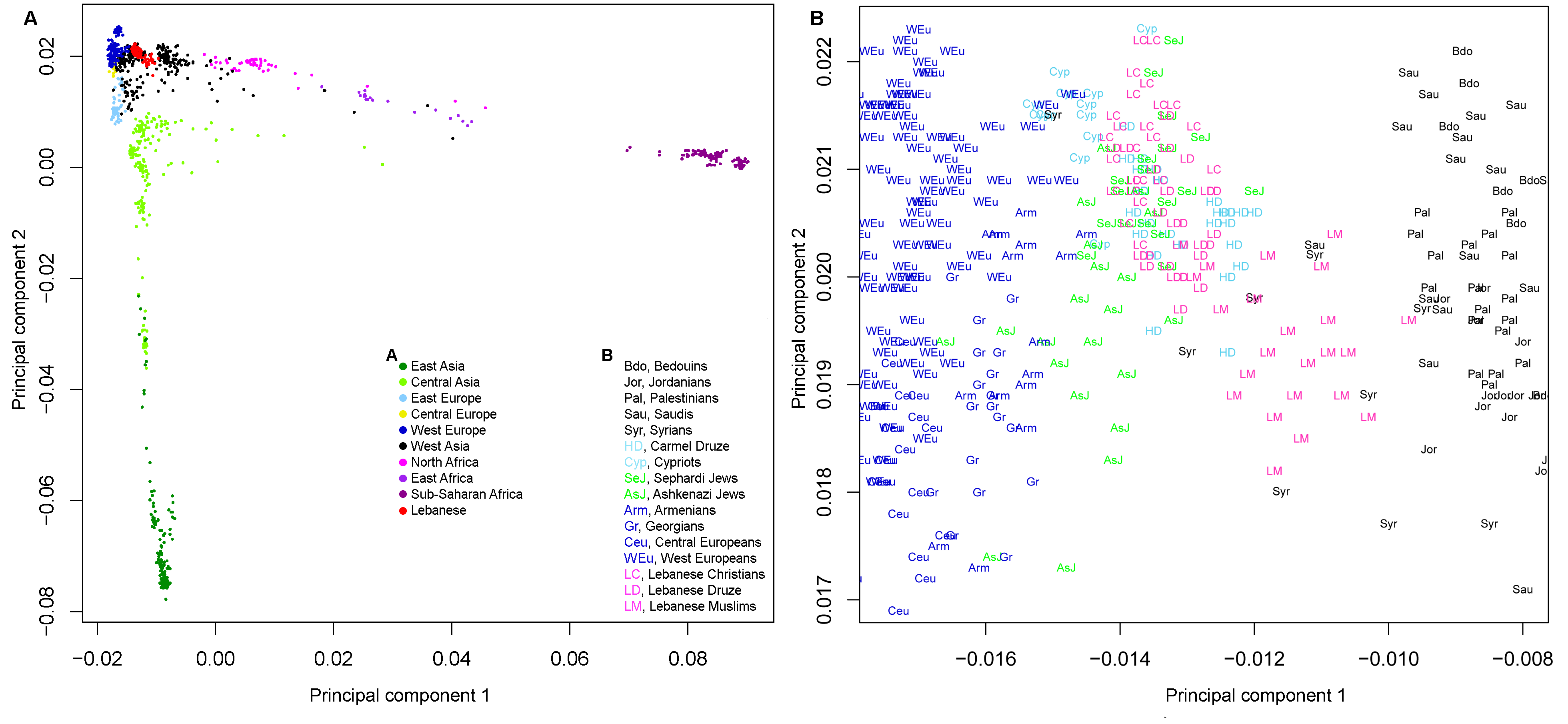

Supplement: Figure S2 — Principle component analysis of >240K SNPs showing the top two components. A) Plot shows global diversity using 50 populations. B) Magnification of West Asia region showing the Levantine populations in their regional and religion context. (TIF) [file pgen.1003316.s002.tif]

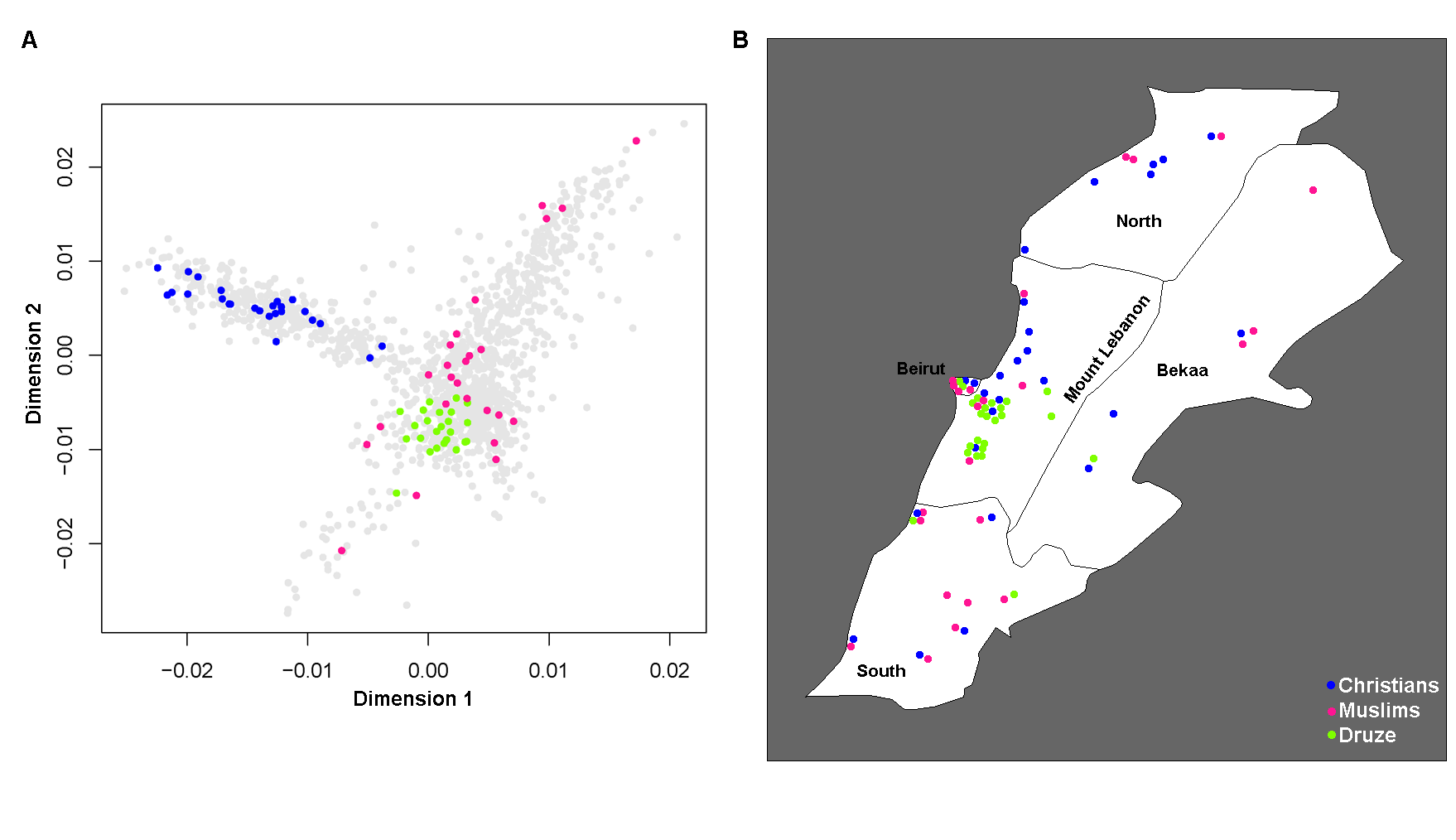

Supplement: Figure S3 — Stratified random sampling of 75 Lebanese samples. A) 25 samples from each of the three main religion groups in Lebanon were randomly chosen from the 1,341 samples illustrated in Figure 1. B) Map of Lebanon showing the distribution of the samples. (TIF) [file pgen.1003316.s003.tif]

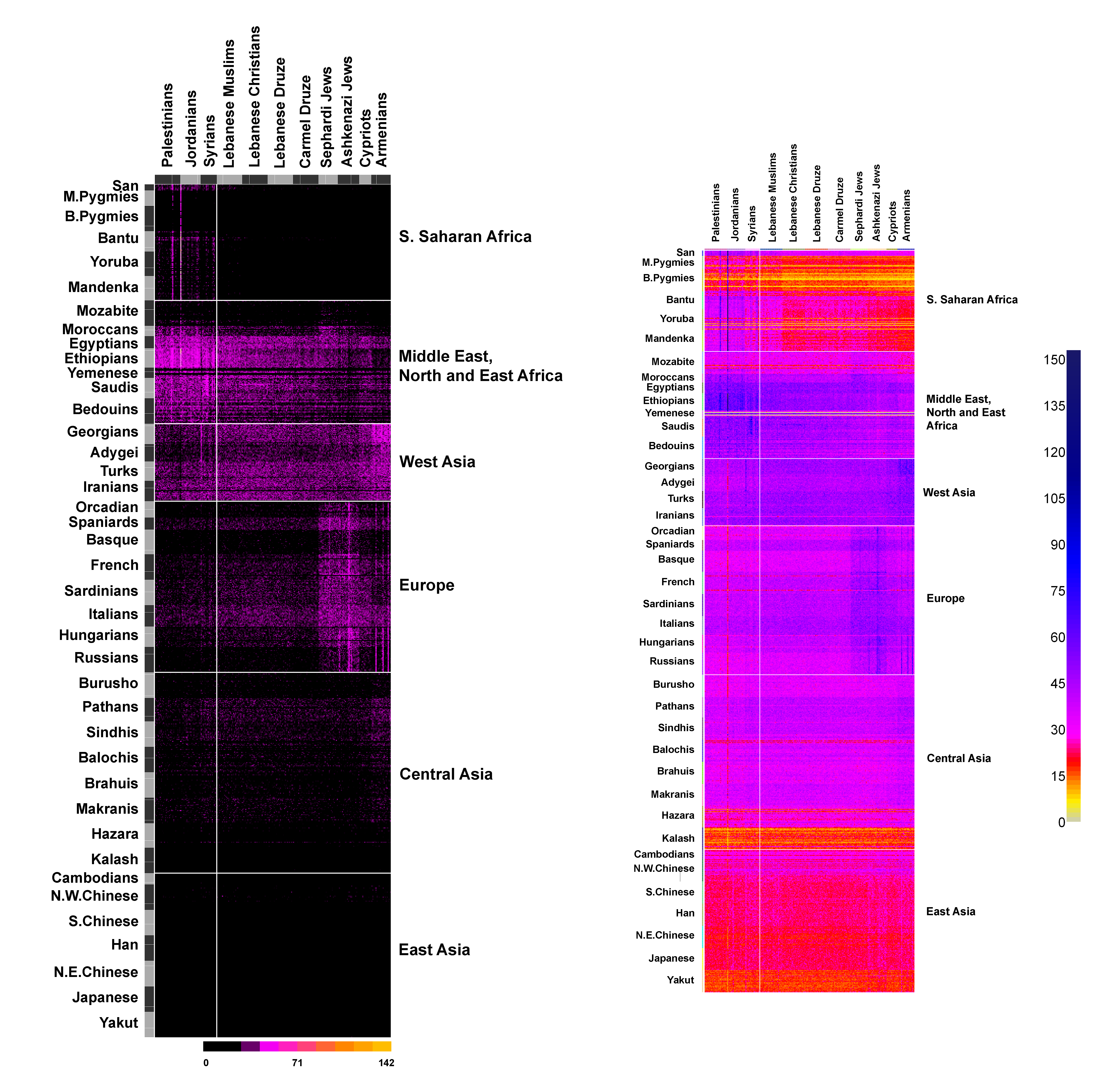

Supplement: Figure S4 — Raw coancestry matrix shows relationships between the Levantines and the world populations. A) Intensity of the colors reflects the number of haplotype chunks donated to the Levantines. The vertical line is a visual aid to reflect the Levantine split observed in the tree. Horizontal lines distinguish the major geographic regions. B) coancestry matrix with an alternative color scale. (TIF) [file pgen.1003316.s004.tif]

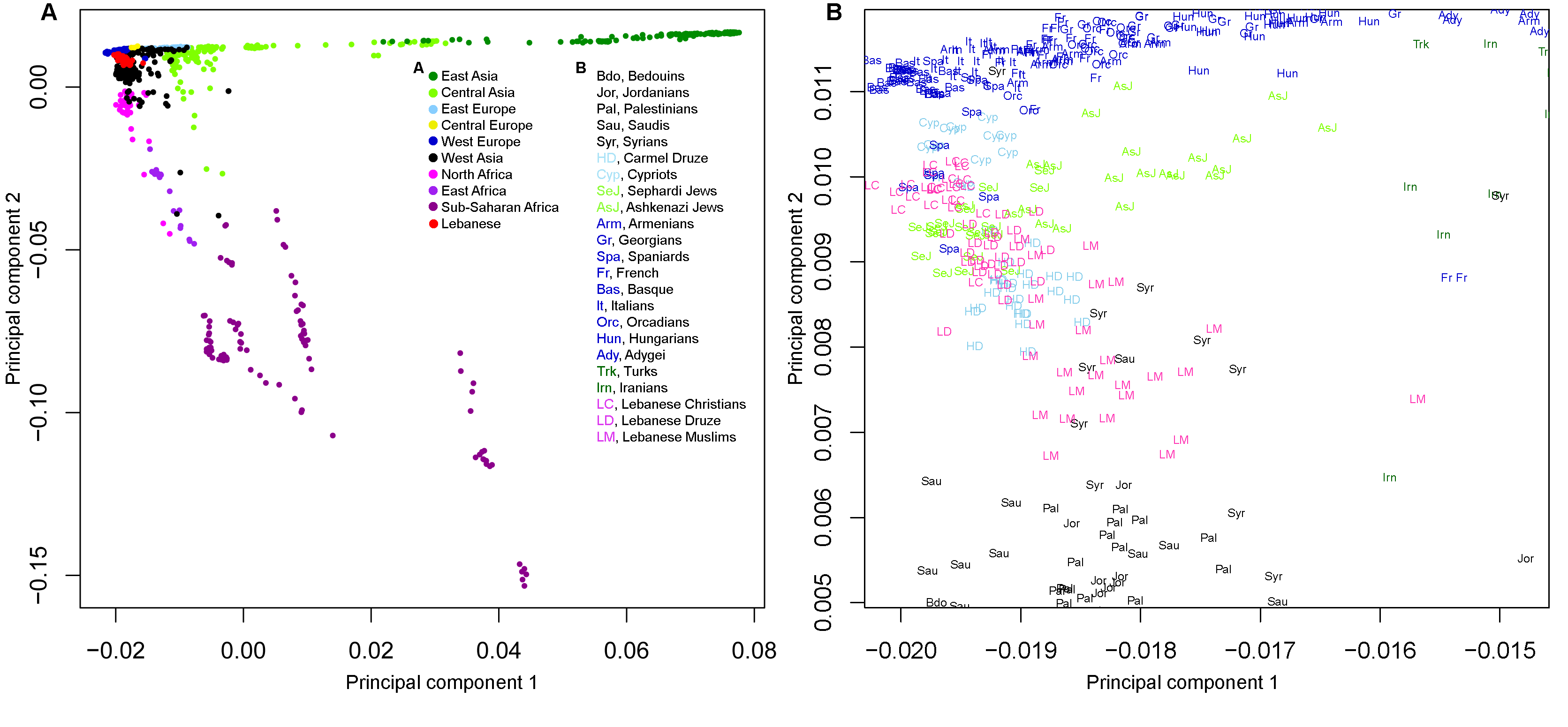

Supplement: Figure S5 — Principle component analysis generated with fineSTRUCTURE using ChromoPainter's coancestry matrix showing the top two components. A) Plot shows global diversity using 50 populations. B) Magnification of West Asia region showing the Levantine populations in their regional and religion context. (TIF) [file pgen.1003316.s005.tif]

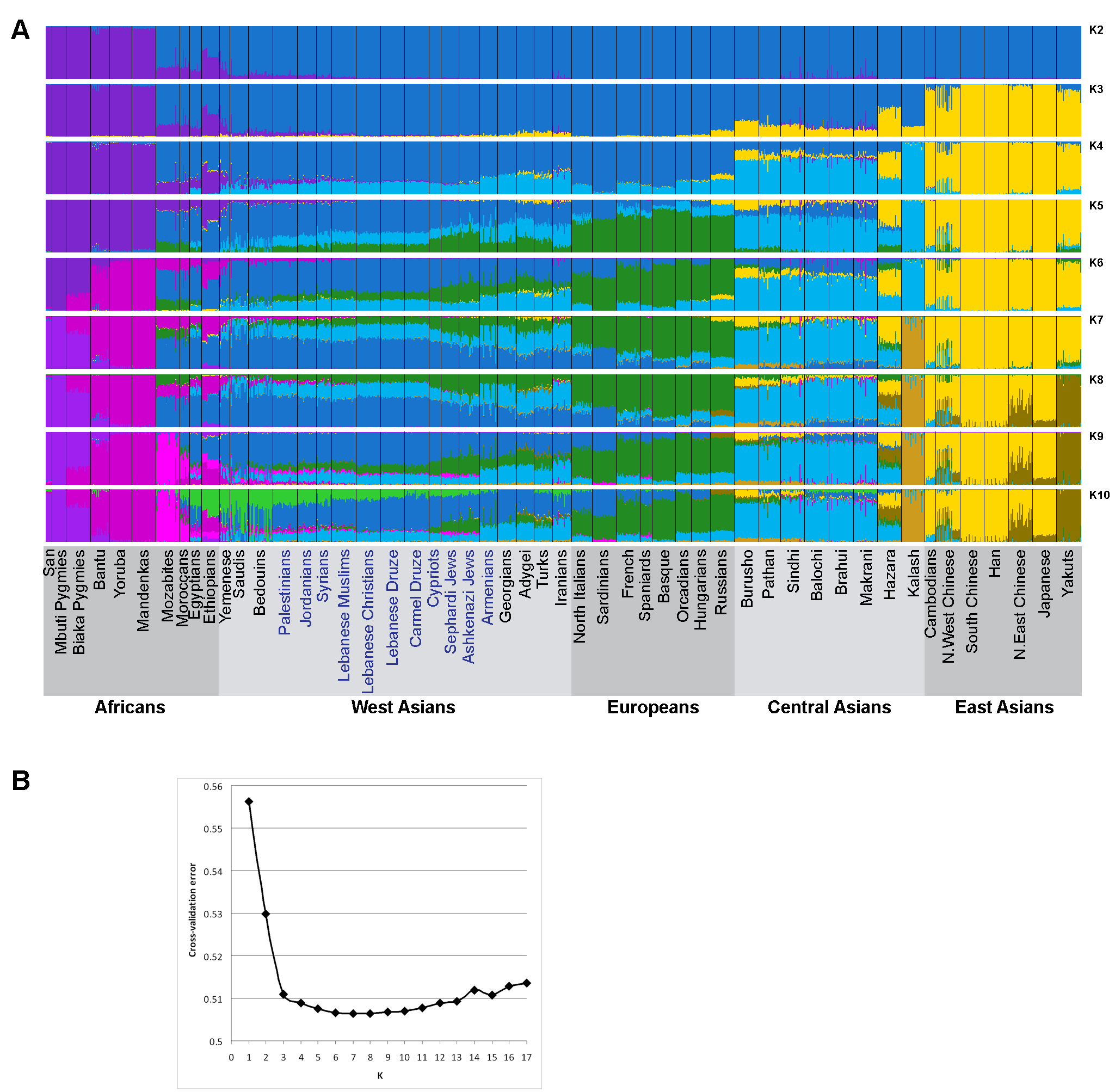

Supplement: Figure S6 — World population structure inferred by ADMIXTURE analysis of >240K autosomal SNPs. A) Each horizontal line represents ancestry probabilities of an individual in 2–10 constructed ancestral populations. Levantine population names are shown in blue. B) Cross-validation plot for the world dataset. (TIF) [file pgen.1003316.s006.tif]
