# Supplementary material for: Genome-Wide Diversity in the Levant Reveals Recent Structuring by Culture
Source: PLoS Genet. 2013 Feb 28;9(2):e1003316. doi: 10.1371/journal.pgen.1003316 (PMC3585000; doi:10.1371/journal.pgen.1003316)
Supplement: Text S1 — Description of the ROLLOFF analysis. (PDF) [file pgen.1003316.s010.pdf]

Text S1 ROLLOFF analysis

PCA Analysis, core clusters, and outlier removal

In order to study the relationship of the Lebanese groups with Europeans and sub-Saharan African populations, we performed PCA [1] with Sardinians, Georgians, Ethiopians and Yoruba [2-4]. We projected the Lebanese samples onto the PCs built using European and sub-Saharan African groups. Figures 1 and 2 show that Lebanese Druze and Christians are homogeneous in their relationship to Europeans and sub-Saharan Africans. The Lebanese Muslims are a very heterogenous group and so we divide these samples into 5 groups based on their position on PC1. In addition, we removed 13 outliers using the outlier removal flag in *smartpca*, part of the EIGENSOFT 3.0 package [1] from Lebanese Druze (n = 6) and Christians (n = 7) as they do not cluster with the bulk of the samples from those groups.

ROLLOFF

In order to estimate the date of the sub-Saharan African ancestry in the Lebanese groups, we performed ROLLOFF [5] analysis using Georgians and Ethiopians as the reference populations. Results are shown in Table 1 and Figure 3. To test that the ROLLOFF analysis is robust to the reference populations used, we ran ROLLOFF using Sardinians and Yoruba as the reference and obtained qualitatively similar results (Table 1 and Figure 4).

Table 1: Dates of sub-Saharan admixture in Lebanese groups

Admixed Group	Number of Samples	Estimated date ± standard error
**References: Georgians and Ethiopians**		
Lebanese Christians	336	88 ± 7
Lebanese Druze	85	46 ± 5
Lebanese Muslims - gr1	7	16 ± 3
Lebanese Muslims - gr2	17	15 ± 3
Lebanese Muslims - gr3	91	19 ± 1
Lebanese Muslims - gr4	747	26 ± 1
Lebanese Muslims - gr5	45	55 ± 8
**References: Sardinians and Yoruba**		
Lebanese Christians	336	95 ± 7
Lebanese Druze	85	38 ± 4
Lebanese Muslims - gr1	7	14 ± 2
Lebanese Muslims - gr2	17	15 ± 2
Lebanese Muslims - gr3	91	20 ± 1
Lebanese Muslims - gr4	747	26 ± 1
Lebanese Muslims - gr5	45	39 ± 5

PCA Projection: PCA Sardinians, Georgians, Yoruba and Ethiopians

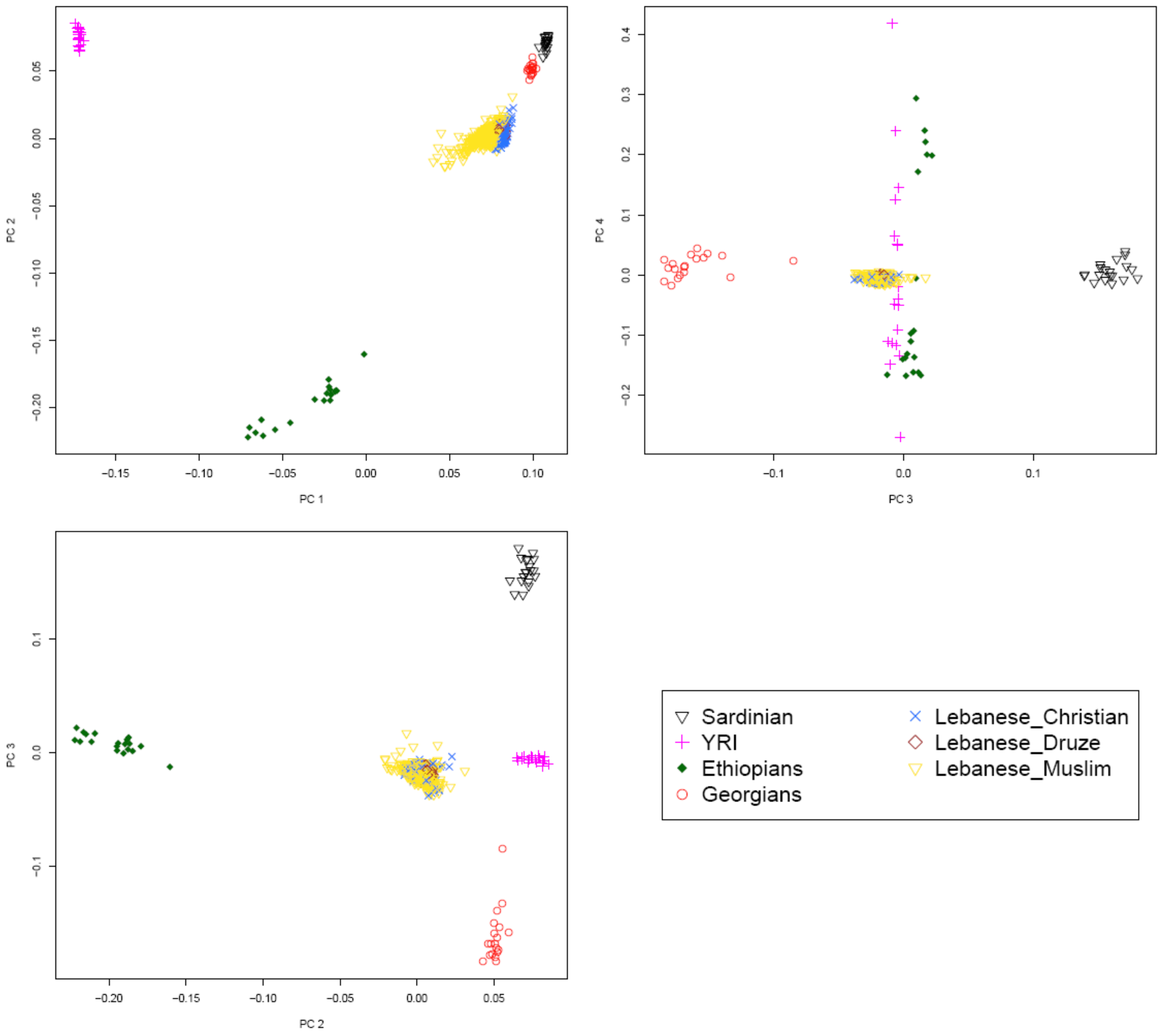

Figure 1: Principal Components Analysis

PCA Loadings for Lebanese Groups

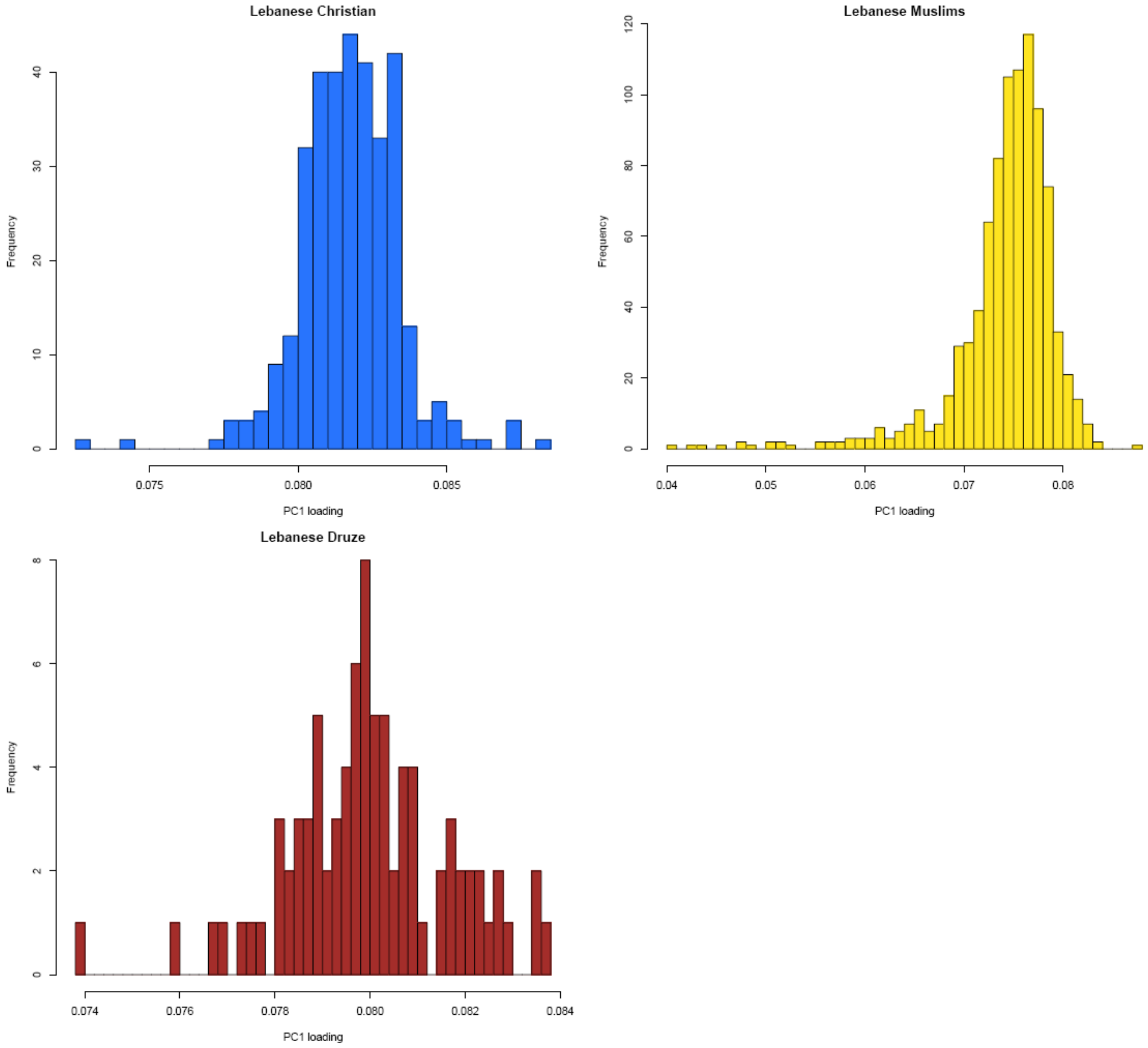

Figure 2: Histogram of PC1 loadings from the PCA Projection of Figure 1

ROLLOFF analysis using Georgians and Ethiopians as references

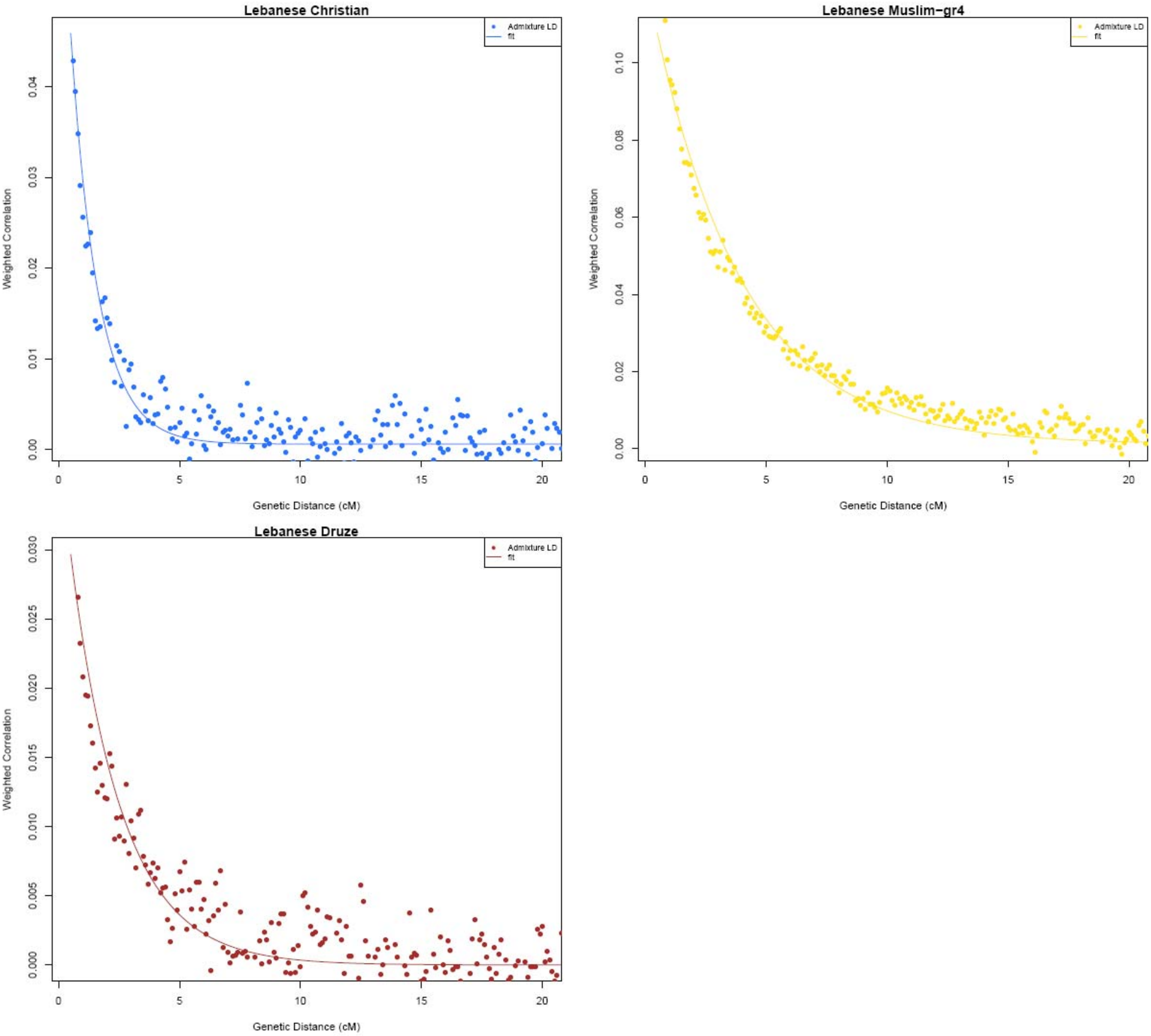

Figure 3: We performed ROLLOFF Analysis using Georgians and Ethiopians as the reference populations. The estimated dates of mixture for Lebanese Christian (in blue), Lebanese Druze (in brown) and Lebanese Muslims (group 4 - in gold) are 88±7, 46±5 and 26±1 respectively.

ROLLOFF analysis using Sardinians and Yoruba as references

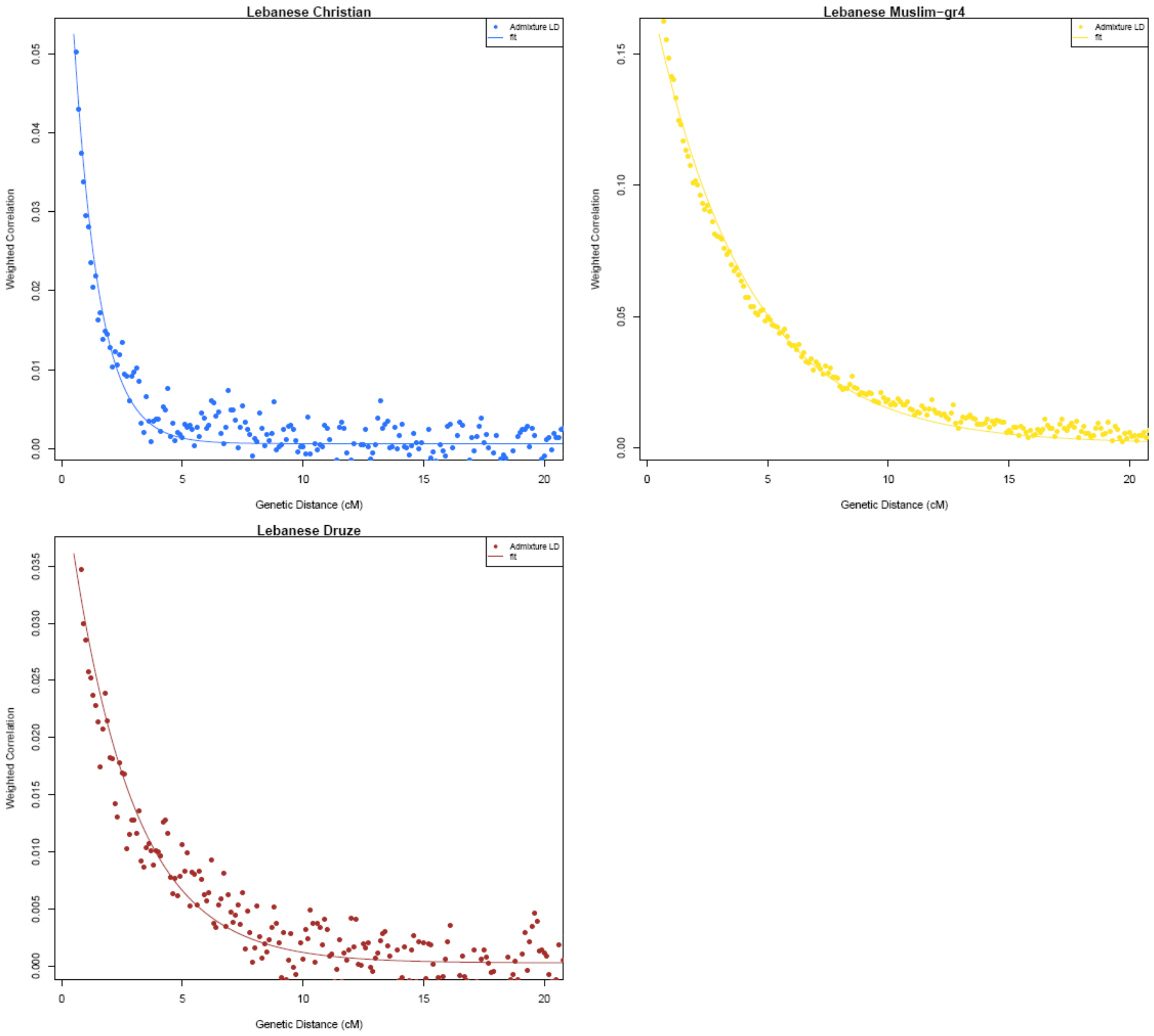

Figure 4: We performed ROLLOFF Analysis using Sardinians and Yoruba as the reference populations. The estimated dates of mixture for Lebanese Christian (in blue), Lebanese Druze (in brown) and Lebanese Muslims (group 4 - in gold) are 95±7, 38±4 and 26±1 respectively.

References

1. Patterson N, Price AL, Reich D (2006) Population structure and eigenanalysis. PLoS Genet 2: e190.
2. The International HapMap Consortium (2005) A haplotype map of the human genome. Nature 437: 1299-1320.
3. Behar DM, Yunusbayev B, Metspalu M, Metspalu E, Rosset S, et al. (2010) The genome-wide structure of the Jewish people. Nature 466: 238-242.
4. Li JZ, Absher DM, Tang H, Southwick AM, Casto AM, et al. (2008) Worldwide human relationships inferred from genome-wide patterns of variation. Science 319: 1100-1104.
5. Moorjani P, Patterson N, Hirschhorn JN, Keinan A, Hao L, et al. (2011) The history of African gene flow into Southern Europeans, Levantines, and Jews. PLoS Genet 7: e1001373.
